# Effects of compound small peptides of Chinese medicine on intestinal immunity and cecal intestinal flora in CTX immunosuppressed mice

**DOI:** 10.3389/fmicb.2022.959726

**Published:** 2022-07-25

**Authors:** Yuqing Cui, Lu Zhang, Chunyu Lu, Mengmeng Dou, Yulan Jiao, Yongzhan Bao, Wanyu Shi

**Affiliations:** ^1^College of Veterinary Medicine, Institute of Traditional Chinese Veterinary Medicine, Hebei Agricultural University, Baoding, China; ^2^Research and Development Department, Ringpu (Baoding) Biological Pharmaceutical Co., Ltd, Baoding, China; ^3^Hebei Veterinary Biotechnology Innovation Center, Hebei Agricultural University, Baoding, China; ^4^Pharmacoefficacy Laboratory, Hebei Provincial Engineering Center for Chinese Veterinary Herbal Medicine, Baoding, China

**Keywords:** Chinese medicine, immune, mice, peptide, small intestine

## Abstract

The study was designed to explore the improvement effect of CSPCM (compound small peptide of Chinese medicine) on intestinal immunity and microflora through the treatment of different doses of CSPCM. A total of 100 male Kunming mice were weighed and divided into five groups, namely, group A (control group), group B (model group), group C (0.1 g/kg·bw CSPCM), group D (0.2 g/kg·bw CSPCM), and group E (0.4 g/kg·bw CSPCM). The use of CTX (cyclophosphamide) caused a series of negative effects: the secretion of IL-2, IL-22, TNF-α, sIgA, length of the villi, and the area of Pey's node were significantly reduced (*P* < 0.05); the depth of crypt and the percent of CD3^+^ and CD4^+^ cells were significantly increased (*P* < 0.05); the cecal flora taxa decreased; the abundance of *Firmicutes* and *Lactobacillus* increased; and the abundance of *Bacteroidetes, Deferribacteres, Proteobacteria, Mucispirillum, Bacteroides*, and *Flexisprra* decreased. The addition of CSPCM improved the secretion of cytokines and the development of intestinal villi, crypts, and Pey's node. The number of CD3^+^ and CD4^+^ cells in groups C, D, and E was significantly higher than that in group B (*P* < 0.05). Compared with group B, the abundance of *Firmicutes* in groups C, D, and E was decreased, and the *Bacteroidetes, Deferribacteres*, and *Proteobacteria* increased. The abundance of *Lactobacillus* decreased, while that of *Mucispirillum, Bacteroides*, and *Flexisprra* increased. It is concluded that cyclophosphamide is extremely destructive to the intestinal area and has a great negative impact on the development of the small intestine, the intestinal immune system, and the intestinal flora. The CSPCM can improve the negative effects of CTX.

## Introduction

Due to the influence of food safety issues, environmental pollution, social pressure, obesity, and antibiotics, human beings are in a subhealthy state (Chakraborty, 2019). The subhealthy state of the human body will lead to the disorder or weakening of the human immune system. At this time, if you are infected with a virus or bacteria, the human immune system cannot accurately identify the pathogen and eliminate it. In addition, the probability of human beings suffering from tumors increases year by year and tends to be younger (Adolescent Young Adult Cancer Collaborators, 2021; Saab, 2021). As an important cancer treatment drug, cyclophosphamide has an unquestionable effect, but the side effects of tumor treatment are also very obvious, the most important of which is the destruction of the immune system (Liu et al., 2021; Zhou et al., 2021). Cyclophosphamide is absorbed by the body and is metabolized in the liver to produce active metabolites to play a role. It plays a role in tumor treatment by inhibiting the function of the immune system (Gomez-Figueroa et al., 2021). After cancer treatment, the treatment of the body no longer suppresses immunity but reshapes the body's immune system. As one of the most important components of complementary and alternative medicines, traditional Chinese medicines (TCM) have been practiced in China and surrounding areas for thousands of years. With rich experiences in fighting diseases and the growing trend of acceptance of complementary and alternative medicines, studies have shown that polysaccharides from *Lycium barbarum* could regulate immune response depending on the modulation of gut microbiota (Ding et al., 2019); ginseng-astragalus-oxymatrine injection could ameliorate cyclophosphamide-induced immunosuppression in mice (Li et al., 2021). Whether it is used alone or in combination, traditional Chinese medicine can effectively play a therapeutic or healthcare effect. The prescription can increase the site of action and exert the effect better.

In animals, the intestine is a special organ that communicates with the outside world. The intestinal immune system can not only accurately identify the survival of the normal flora in the intestine but also ensure that the bacteria in the intestine will not break through the barrier in the intestine and enter other organs of the body (Dominguez-Bello et al., 2019). The intestine is a complex ecosystem consisting of the intestinal epithelium, immune cells, mucus layer, and microbial communities (Jiao et al., 2020). The intestinal immune system can balance the immune response to pathogens and intestinal flora under normal conditions. Recent studies show that the intestinal flora is an important factor in stimulating the immune system (Kishida et al., 2018), as indicated by the fact that germ-free mice have poorly developed lymphoid tissues, spleens with few germinal centers, and poorly formed T and B cell zones, hypoplastic Pey's node, lower numbers of lamina propria CD4+ cells and IgA-producing plasma cells (Macpherson and Haris, 2004), and aberrant development and maturation of isolated lymphoid follicles (Bouskra et al., 2008). The intestinal microbiome has been considered a new method for the treatment of various intestinal diseases. Chinese herbal medicine interacts with intestinal microorganisms: the toxic substances and substances that cannot be directly absorbed in Chinese herbal medicine are metabolized and decomposed by intestinal microorganisms. For example, the intestinal flora degrades cinnabar into nontoxic mercuric polysulfides (Zhou et al., 2011). Human intestinal bacteria could convert aconitine to lipoaconitine (Feng et al., 2019). Traditional Chinese medicine and its metabolites affect the composition and metabolism of intestinal microbes. Astragalus administration significantly increased gut microbiota richness and diversity and significantly altered the abundance of several bacterial taxa, inducing an increased abundance of *Lactobacillus* and *Bifidobacterium* (Li et al., 2019). Red ginseng could alleviate *Escherichia coli*-induced gut dysbiosis (Han et al., 2020). The traditional theory of traditional Chinese medicine emphasizes that “both over and under are diseases.” A healthy animal body needs to achieve two balances in the intestinal tract: the balance between the intestinal flora and the animal body and the balance between various intestinal bacteria. When either of these balances is disrupted, the animal's body becomes dysfunctional.

Traditional Chinese herbal medicine (CHM) has evolved for thousands of years in China and still plays an important role in animal health. Since Brantl et al. (1979) initially isolated a novel opioid peptide from the bovine casein peptone in 1979, academic circles have been challenging the traditional concepts of protein nutrition by discovering the existence of bioactive peptides from plant and animal proteins (Liu et al., 2018). As part of the active ingredients of CHM, peptides have attracted the long-term enthusiasm of researchers (Cui et al., 2021). Peptides are widely distributed in plants and animals and have various pharmacological activities and potential medicinal values (Ling et al., 2018; Liu et al., 2018). Soy peptide is more easily absorbed than soy protein and is related to the maintenance, reinforcement, or restoration of the intestinal barrier function (Kimura and Arai, 1988). Walnut peptide intake augmented the antioxidant defense system and accelerated the survival rate (Zhu et al., 2019). Mice were treated with CSPCM containing 64.8% soy peptide, 25% wheat germ powder, 10% astragalus hydrolysate, and 0.2% vitamin C. Peptides are amino acids linked by amide bonds to compounds in the middle of amino acids and proteins. Small peptides are compounds of 2–3 amino acids linked together. Some di-/tripeptides permeate through the intestinal membranes in their intact forms *via* peptide transporter systems (Shen and Matsui, 2017). Compared with free amino acids, small peptides have the advantages of fast absorption, low energy consumption, and a high absorption rate (Matthews, 1975). They have independent absorption mechanisms in the animal body and do not interfere with each other (Webb Ke and Matthews, 1993). Making a traditional Chinese medicine into a peptide can increase its absorption rate while retaining its potency. In this study, mice were tested with CSPCM (containing 64.8% soybean peptide, 25% wheat germ powder, 10% astragalus hydrolysate, and 0.2% vitamin C) to explore the effects of CSPCM on intestinal immunity and intestinal microflora in human immunosuppressed state.

## Materials and methods

### Chemicals

The CSPCM was provided by HeBei TaiFeng Biotechnology Co., Ltd. The manufacturing process of CSPCM used in this experiment is as follows: confirming the ratio of material to water → adding enzyme at high temperature → rotating oriented enzyme → microfiltration → active purification of traditional Chinese medicine → recombination of active peptide → low-temperature concentration → spray drying. The acid-soluble protein content was detected after the preparation, and the acid-soluble protein was required to account for more than 30% of the total protein. CTX was purchased from Source Leaf Biotechnology Co., Ltd. (Shanghai, China). Hematoxylin dyeing solution and eosin were purchased from Beijing Boaotuo Technology Co., Ltd. (Beijing, China). Anti-stripping glass slides and cover glass were purchased from Shanghai Weibo Biotechnology Co., Ltd. (Shanghai, China). Feather microtome blade R35 was purchased from Leica Microsystems (Shanghai) Trading Co., Ltd. (Shanghai, China). IL-10, IL-17, IL-2, IL-22, TNF-α, and sIgA Elisa kits were purchased from Shanghai Enzyme-Linked Biotechnology Co., Ltd. (Shanghai, China). FITC anti-mouse CD3+ antibody, APC anti-mouse CD4+ antibody, PE anti-mouse CD8+ antibody, True-Nuclear™ Transcription Factor Buffer Set, True Nuclear TM 4X Fix Concentrate, True Nuclear TM 10X Per, and True Nuclear TM Fix Diluent were purchased from BioLegend, Inc (California, USA). DNeasy PowerSoil Kit was purchased from QIAGEN, Inc. (Hilden, Germany). Agencourt AMPure Beads was purchased from Beckman Coulter Co., Ltd. (Indianapolis, USA). PicoGreen dsDNA Assay Kit was purchased from Invitrogen Co., Ltd. (Carlsbad, USA).

### Animals and experimental design

Male-specific pathogen-free (SPF) Kunming mice weighing 20.0 ± 2.0 g (6–8 weeks) were purchased from Spfanimals (Beijing) Laboratory Animal Science and Technology Co., Ltd. (Beijing, China). All mice were provided specific pathogen-free food and water *ad libitum* and acclimated for 1 week. All animal studies were approved by the Ethics Committee of Animal Experiments of Heibei Agricultural University.

After 1 week of adaptation, 100 male Kunming mice were divided into 5 groups, namely, group A (control group), group B (model group), group C (0.1 g/kg·bw CSPCM), group D (0.2 g/kg·bw CSPCM), and group E (0.4 g/kg·bw CSPCM). From day 1 to day 3, all groups except A were intraperitoneally injected with 0.08 g/kg·bw CTX, and the A group was intraperitoneally injected with normal saline. From the fourth day of the experiment, the A group and B group were given oral normal saline intragastrally, and the C, D, and E groups were given 0.1, 0.2, and 0.4 g/kg·bw CSPCM for 14 days, respectively. After 14 days of gavage, the middle section of the small intestine was dissected longitudinally, rinsed with normal saline, and then stored in a refrigerator at −80°C for use. Mesenteric lymph nodes were collected for flow cytometry. Cecal contents were taken and placed in cryopreservation tubes, preserved in dry ice, and transported to Paiseno Biotechnology Co., Ltd., for 16S rRNA high-throughput sequencing.

### Cytokines in small intestinal tissue and morphological observation of small intestine

A 10% tissue homogenate was prepared from the cleaned midsection of the small intestine, and an ELISA kit was used for detection. Sections of the small intestine were taken, and the contents were gently rinsed with normal saline and soaked in a 3% formaldehyde solution. The sections of the small intestine and Pey's node were prepared by paraffin section and observed with a light microscope after HE staining. The length of villi, crypt depth, and Pey's node area of the small intestine were measured using the Image J software.

### Flow cytometry

After grinding the mesenteric lymph nodes to extract the cells and washing with PBS, the cell concentration was adjusted to 100 cells/L. The corresponding antibodies were added according to the instructions and incubated at 4°C in the dark for 30 min. A volume of 500 μl of PBS containing 1% paraformaldehyde was added and the solution was tested using the machine.

### High-throughput sequencing of cecal flora 16S rRNA

This experiment used 16S rRNA V3V4 high-throughput sequencing, and the sequencing primer sequence was F:ACTCCTACGGGAGGCAGCA R:GGACTACHVGGGTWTCTAAT. 16S rRNA sequencing platform was Illumina MiSeq platform (Shanghai Personal Biotechnology Co., Ltd.). Preliminary screening of the original off-machine data was performed for high-throughput sequencing based on sequence quality; retesting and supplementary testing of problem samples were also performed. Through depriming, splicing, quality filtering, deduplication, de-chimerism, and clustering, the original sequence was divided into the library and the sample according to the index and barcode information, and the barcode sequence was removed. After obtaining the OTU representative sequence, statistics on its length distribution were performed to check whether the length of these sequences is equivalent to the length range of the sequenced target fragments, whether there are sequences of abnormal length, etc. At the level of species taxonomic composition, through various unsupervised and supervised sorting, clustering, and modeling methods, combined with corresponding statistical testing methods, we can further measure the differences in species abundance composition between different samples (groups), and try to find symbol species. The sequencing depth was 95% of the minimum sample sequence size. In this experiment, the minimum sequence number was 36,652, and the sequencing depth was 81,962.

### Statistical analysis

All of the experimental data are presented as mean ± standard deviation (SD). The results were analyzed using the IBM SPSS Statistics 19 software (IBM Inc., Chicago, IL, USA) for single-factor analysis. A value of *P* < 0.05 was regarded to be statistically significantly different.

## Results

### Effects of CSPCM on the levels of cytokines in small intestinal tissue

As shown in Table 1, compared with group A, IL-2, IL-22, TNF-α, and sIgA in group B significantly decreased (*P* < 0.05); IL-10, IL-2, IL-22, TNF-α, and sIgA in group E significantly increased (*P* < 0.05); TNF-α in group D significantly increased (*P* < 0.05). Compared with group B, IL-2, IL-22, TNF-α, and sIgA in group C significantly increased (*P* < 0.05); IL-17, IL-2, IL-22, TNF-α, and sIgA in group D significantly increased (*P* < 0.05); IL-10, IL-17, IL-2, IL-22, TNF-α, and sIgA in group E significantly increased (*P* < 0.05).

**Table 1:** Effects of CSPCM on the levels of cytokines in small intestinal tissue.

**Group**	**A**	**B**	**C**	**D**	**E**
IL-10 (pg/ml)	382.0 ± 9.1	380.93 ± 6.45	384.32 ± 33.81	391.79 ± 21.86	435.77 ± 18.23^*#^
IL-17 (pg/ml)	36.17 ± 2.36	34.44 ± 0.72	35.21 ± 2.82	37.73 ± 2.39^#^	38.7 ± 1.32^#^
IL-2 (pg/ml)	301.17 ± 4.55^#^	243.4 ± 11.45*	304.71 ± 3.62^#^	309.64 ± 9.29^#^	323.97 ± 11.56^*#^
IL-22 (pg/ml)	28.25 ± 1.15^#^	26.78 ± 1.19*	28.91 ± 1.47^#^	29.05 ± 0.57^#^	31.7 ± 0.82^*#^
TNF-α (pg/ml)	501.79 ± 24.1^#^	467.73 ± 14*	522.78 ± 27.81^#^	551.33 ± 29.01^*#^	620.48 ± 28.03^*#^
sIgA (μg/ml)	32.43 ± 1.65^#^	30.13 ± 1.39*	31.34 ± 1.34^#^	35.56 ± 2.68^#^	37.73 ± 1.27^*#^

*Significant difference with group A (P <0.05).

*^#^Significant difference with group B (P <0.05), same as in Table 2*.

### Effects of CSPCM on the morphological features of the small intestine

**Figure 1:**
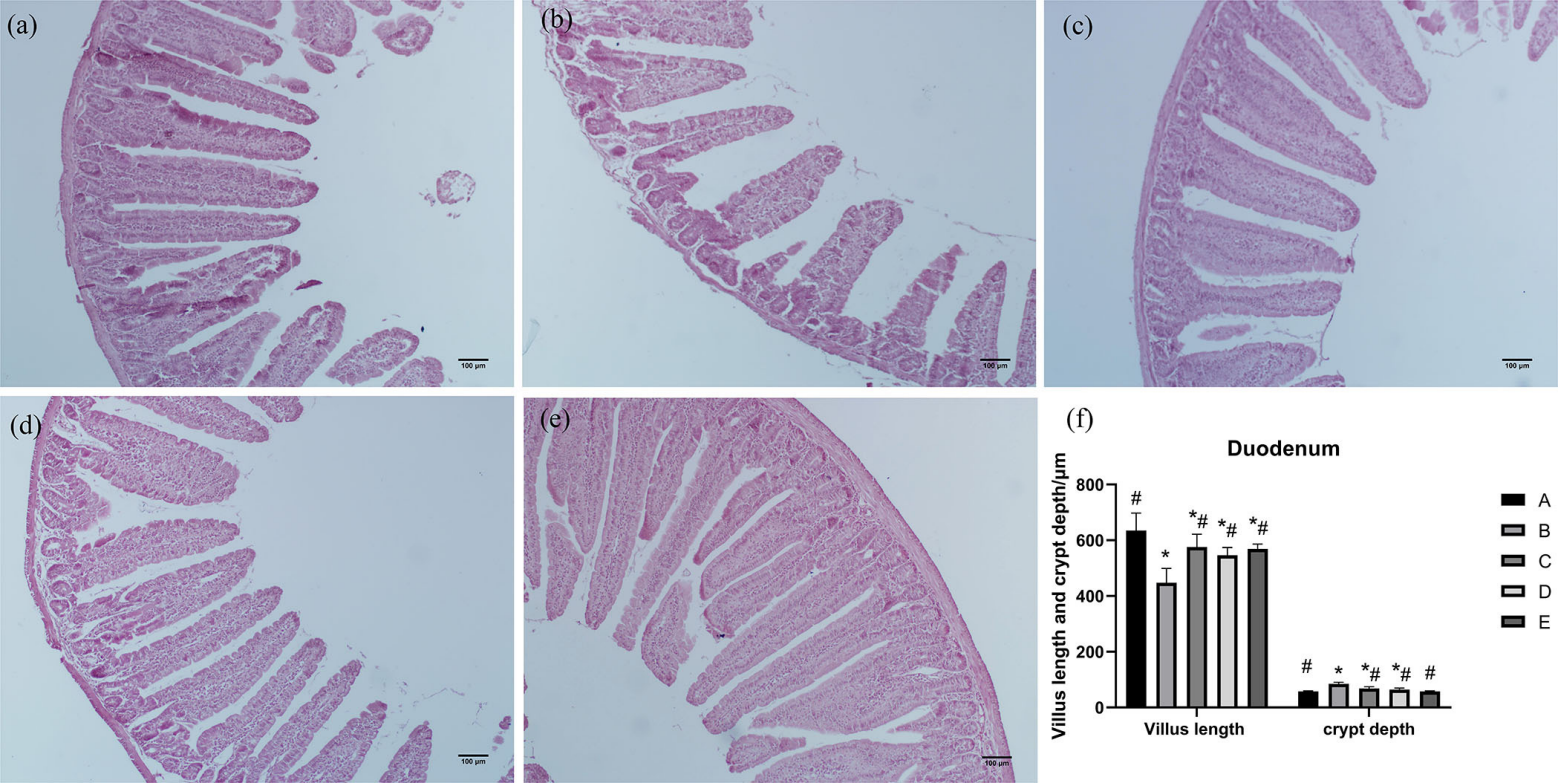
Effects of CSPCM on the morphological features of the duodenum. **(a)** Morphological observation of duodenum in group A. **(b)** Morphological observation of duodenum in group B. **(c)** Morphological observation of duodenum in group C. **(d)** Morphological observation of duodenum in group D. **(e)** Morphological observation of duodenum in group E. **(f)** Effect of CSPCM on duodenal villus length and crypt depth. *Significant difference with group A (*P* < 0.05). ^#^Significant difference with group B (*P* < 0.05).

As shown in Figure 1, duodenal villi were leaf-like. Duodenal villi in groups A, C, D, and E were arranged neatly and closely, while in group B they were sparse and shorter. Compared with group A, villus length of the duodenum in group B was significantly decreased (*P* < 0.05), crypt depth of the duodenum in groups B, C, and D was significantly increased (*P* < 0.05), and villus length of the duodenum in groups C, D, and E was significantly increased (*P* < 0.05). Compared with group B, villus length of the duodenum in groups A, C, D, and E was significantly increased (*P* < 0.05), and villus length and crypt depth of the duodenum in groups A, C, D, and E was significantly decreased (*P* < 0.05).

**Figure 2:**
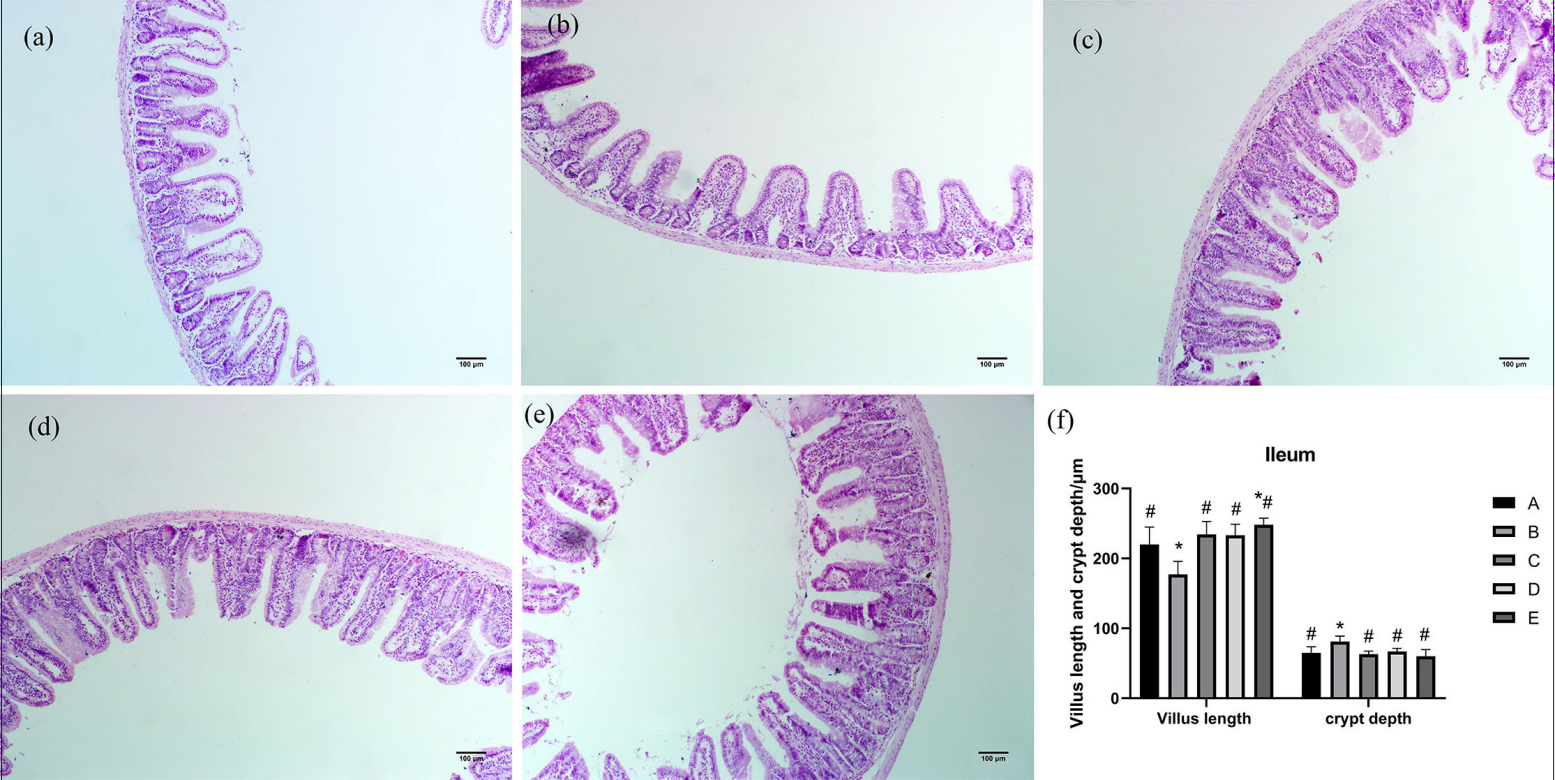
Effects of CSPCM on the morphological features of jejunum. **(a)** Morphological observation of jejunum in group A. **(b)** Morphological observation of jejunum in group B. **(c)** Morphological observation of jejunum in group C. **(d)** Morphological observation of jejunum in group D. **(e)** Morphological observation of jejunum in group E. **(f)** Effect of CSPCM on jejunum villus length and crypt depth. *Significant difference with group A (*P* < 0.05). ^#^Significant difference with group B (*P* < 0.05).

As shown in Figure 2, jejunum villi were mainly finger-like. Morphological observation of the jejunum showed that the villi length in group B was shorter than that in group A. The ileum villi in groups C, D, and E were short, conical, and loosely arranged. Compared with group A, the villus length of jejunum in group B was significantly decreased (*P* < 0.05), villus length of jejunum in groups C, D, and E was significantly increased (*P* < 0.05), and crypt depth of jejunum in group B was significantly increased (*P* < 0.05). Compared with group B, villus length of jejunum in groups A, C, D, and E was significantly increased (*P* < 0.05), and crypt depth of jejunum in groups A, C, D, and E was significantly decreased (*P* < 0.05).

**Figure 3:**
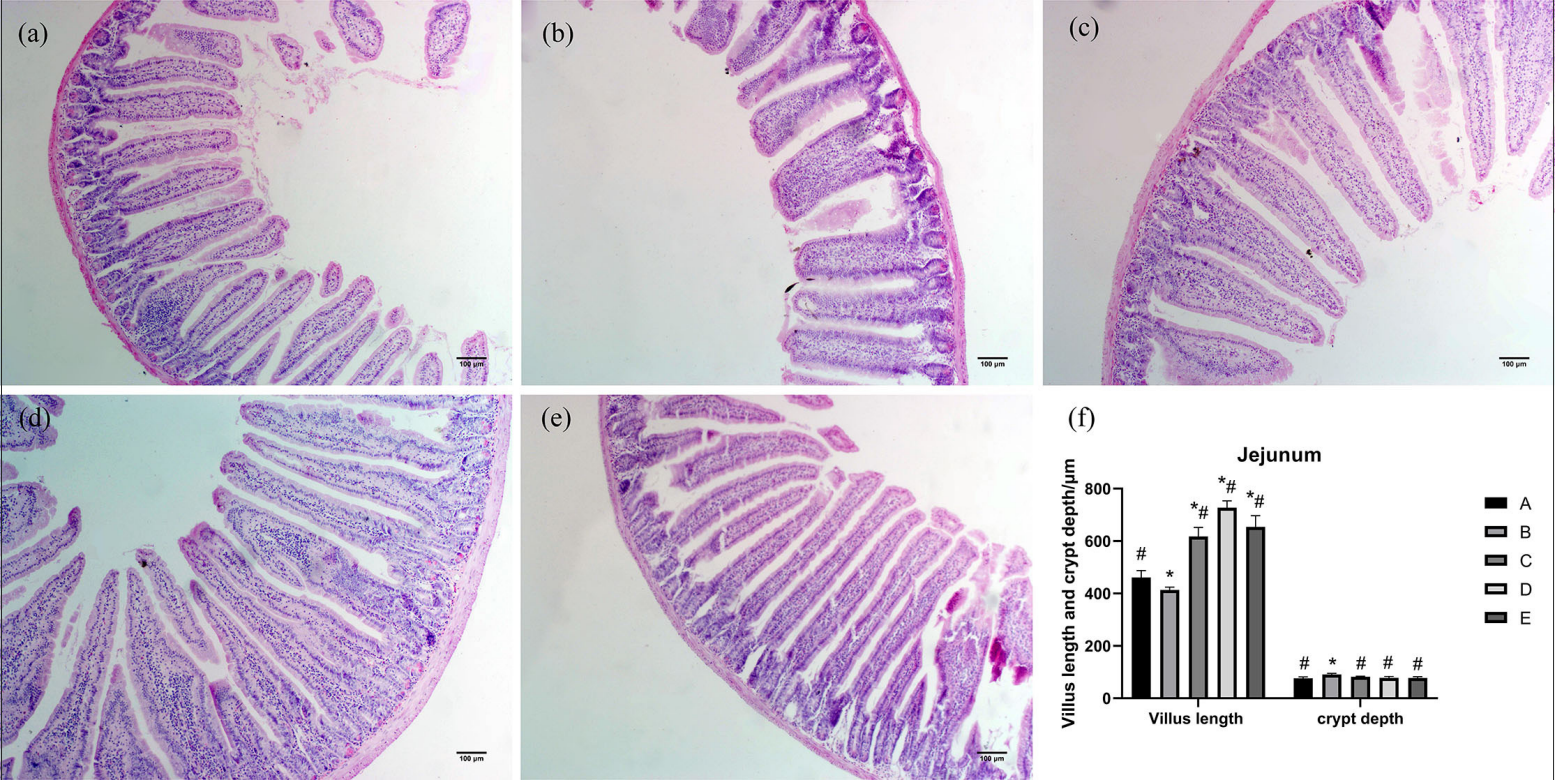
Effects of CSPCM on the morphological features of the ileum. **(a)** Morphological observation of ileum in group A. **(b)** Morphological observation of ileum in group B. **(c)** Morphological observation of ileum in group C. **(d)** Morphological observation of ileum in group D. **(e)** Morphological observation of ileum in group E. **(f)** Effect of CSPCM on ileum villus length and crypt depth. *Significant difference with group A (*P* < 0.05). ^#^Significant difference with group B (*P* < 0.05).

As shown in Figure 3, ileum villi were short, conical, and arranged loosely. The ileum villi in groups C, D, and E were arranged neatly and closely, while the villi in group B were sparse and shorter. Compared with group A, villus length of ileum in group B was significantly decreased (*P* < 0.05), villus length of ileum in group E was significantly increased (*P* < 0.05), and crypt depth of ileum in group B was significantly increased (*P* < 0.05). Compared with group B, villus length of ileum in groups A, C, D, and E was significantly increased (*P* < 0.05), and crypt depth of ileum in groups A, C, D, and E was significantly decreased (*P* < 0.05).

### Effects of CSPCM on the morphological features of Pey's node

**Figure 4:**
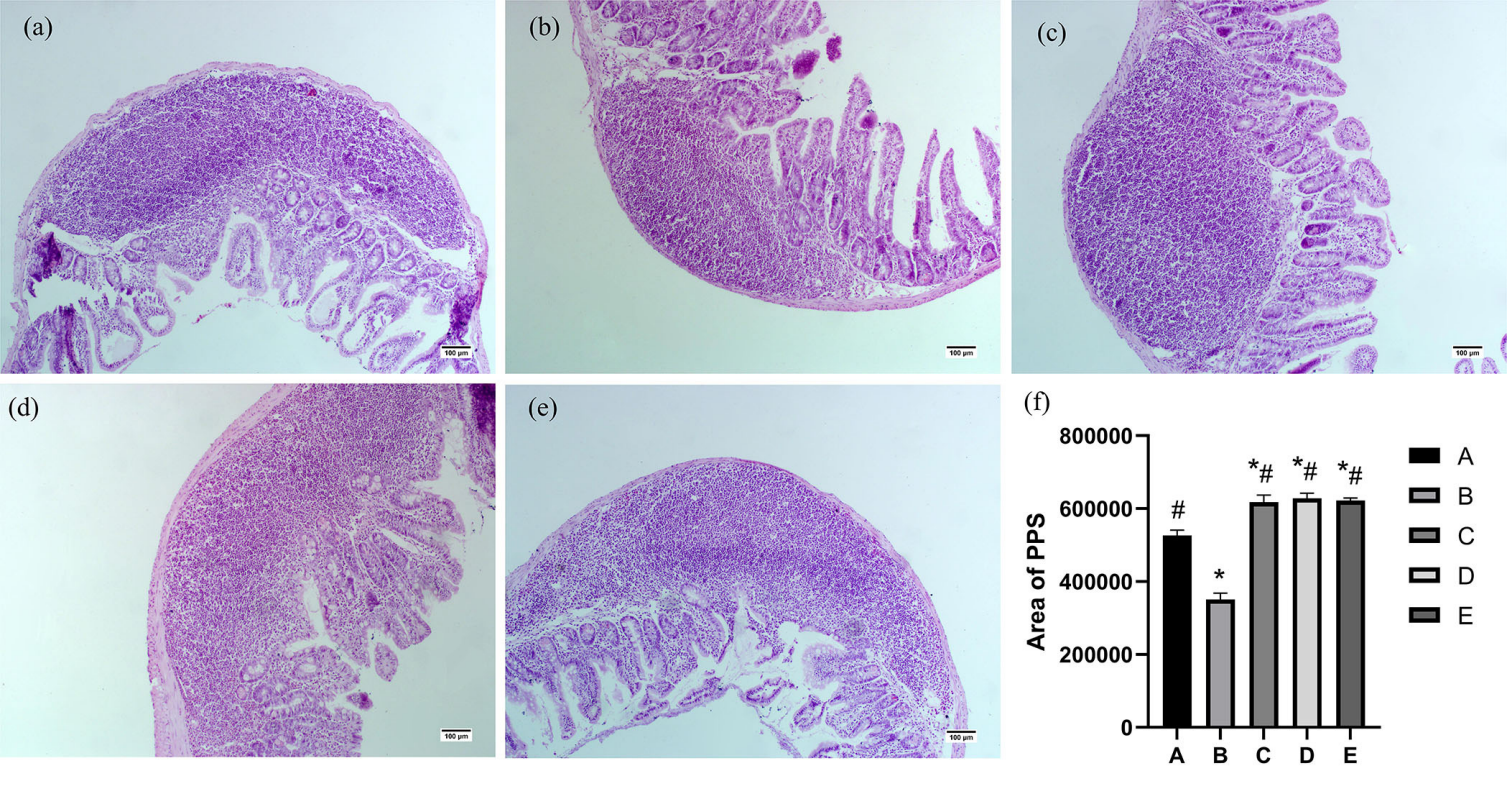
Effects of CSPCM on the morphological features and area analysis of PPS. **(a)** Morphological observation of duodenum in group A. **(b)** Morphological observation of PPS in group B. **(c)** Morphological observation of PPS in group C. **(d)** Morphological observation of PPS in group D. **(e)** Morphological observation of PPS in group E. **(f)** Effects of CSPCM on area analysis of PPS. *Significant difference with group A (*P* < 0.05). ^#^Significant difference with group B (*P* < 0.05).

As shown in Figure 4, the Pey's node area of group B was significantly lower than that of group A (*P* < 0.05). The Pey's node area in groups C, D, and E was significantly higher than that in groups A and B (*P* < 0.05).

### Effects of CSPCM on the percent of CD3+, CD4+, CD8+, and CD4+/CD8+ value in mesenteric lymph nodes

As shown in Table 2, the percentage of CD3+ cells in groups A, C, D, and E was significantly higher than that in group B(*P* < 0.05). The percentage of CD4^+^ cells in groups A, C, D, and E was significantly higher than that in group B (*P* < 0.05). CD4^+^/CD8^+^ in groups A and E was significantly higher than that in group B(*P* < 0.05).

**Table 2:** Effects of CSPCM on CD4+ cells, CD8+ cells, and CD4+/CD8+ in mesenteric lymph nodes.

**Group**	**A**	**B**	**C**	**D**	**E**
CD3+ (%)	98.64 ± 1.34^#^	13.26 ± 2.56*	99.6 ± 0.25^#^	99.77 ± 0.1^#^	99.48 ± 0.11^#^
CD4+ (%)	68.86 ± 4.7^#^	36.65 ± 8.63*	64.39 ± 6.86^#^	57.69 ± 9.39^#^	58.19 ± 9.45^#^
CD8+ (%)	18.18 ± 1.77	17.85 ± 2.84	21.29 ± 6.05	17.18 ± 1.4	14.71 ± 2.67
CD4+/CD8+	3.79 ± 1^#^	2.08 ± 2.49*	3.26 ± 0.81	3.35 ± 1.67	3.97 ± 0.24^#^

### Analysis of the effect of CSPCM on intestinal flora

#### Length distribution of intestinal flora sequence

**Figure 5:**
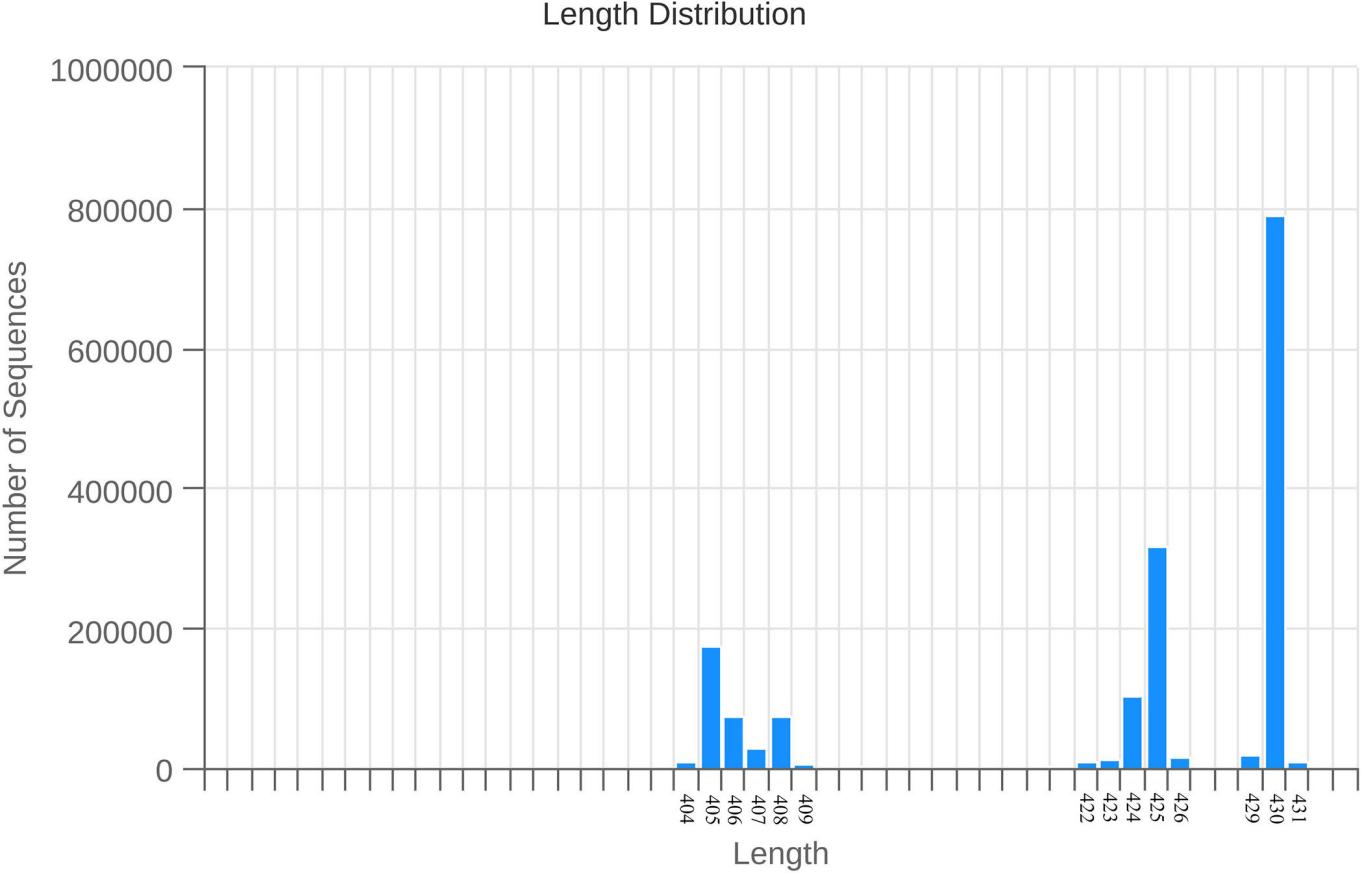
Overall sample sequence length distribution. Note: The abscissa is the length of the sequence, and the ordinate is the number of sequences.

As shown in Figure 5, the number of sequences with a length of 50 is 143, the number of sequences with a length of 192 is 15, the number of sequences with a length of 217 is 3, the number of sequences with a length of 271 is 2, the number of sequences with a length of 273 is 2, and the number of sequences with a length of 278 is 4. The number of sequences with a length of 284 is 6, the number of sequences with a length of 293 is 2, the number of sequences with a length of 318 is 5, the number of sequences with a length of 337 is 10, and the number of sequences with a length of 338 is 12. The number of sequences with a length of 341 is 2, the number of sequences with a length of 343 is 3, the number of sequences with a length of 344 is 2, the number of sequences with a length of 353 is 3, the number of sequences with a length of 369 is 2, and the number of sequences with a length of 385 is 4. The number is 4, the number of sequences with a length of 386 is 2, the number of sequences with a length of 392 is 2, the number of sequences with a length of 393 is 9, the number of sequences with a length of 403 is 2, and the number of sequences with a length of 404 is 8,691. The number of sequences with a length of 405 is 175,621, the number of sequences with a length of 406 is 75,755, the number of sequences with a length of 407 is 27,954, the number of sequences with a length of 408 is 75,828, the number of sequences with a length of 409 is 6,258, and the number of sequences with a length of 410 is 445. The number of sequences with a length of 411 is 2,018, the number of sequences with a length of 412 is 2,841, the number of sequences with a length of 413 is 86, the number of sequences with a length of 414 is 40, the number of sequences with a length of 415 is 116, and the number of sequences with a length of 416 is 73. The number of sequences of length 418 is 12, the number of sequences of length 419 is 36, the number of sequences of length 420 is 2,061, the number of sequences of length 421 is 9,717, and the number of sequences of length 423 is 14,288. The number of sequences with a length of 424 is 103,357, the number of sequences with a length of 425 is 317,111, the number of sequences with a length of 426 is 15,984, the number of sequences with a length of 427 is 3,724, the number of sequences with a length of 428 is 195, and the length of 429 is 197,40. The number of sequences of length is 197,40, the number of sequences of length 430 is 789,995, the number of sequences of length 431 is 8,390, the number of sequences of length 432 is 209, and the number of sequences of length 433 is 9.

#### The effect of CSPCM on species differences and marker analysis in mice samples

**Figure 6:**
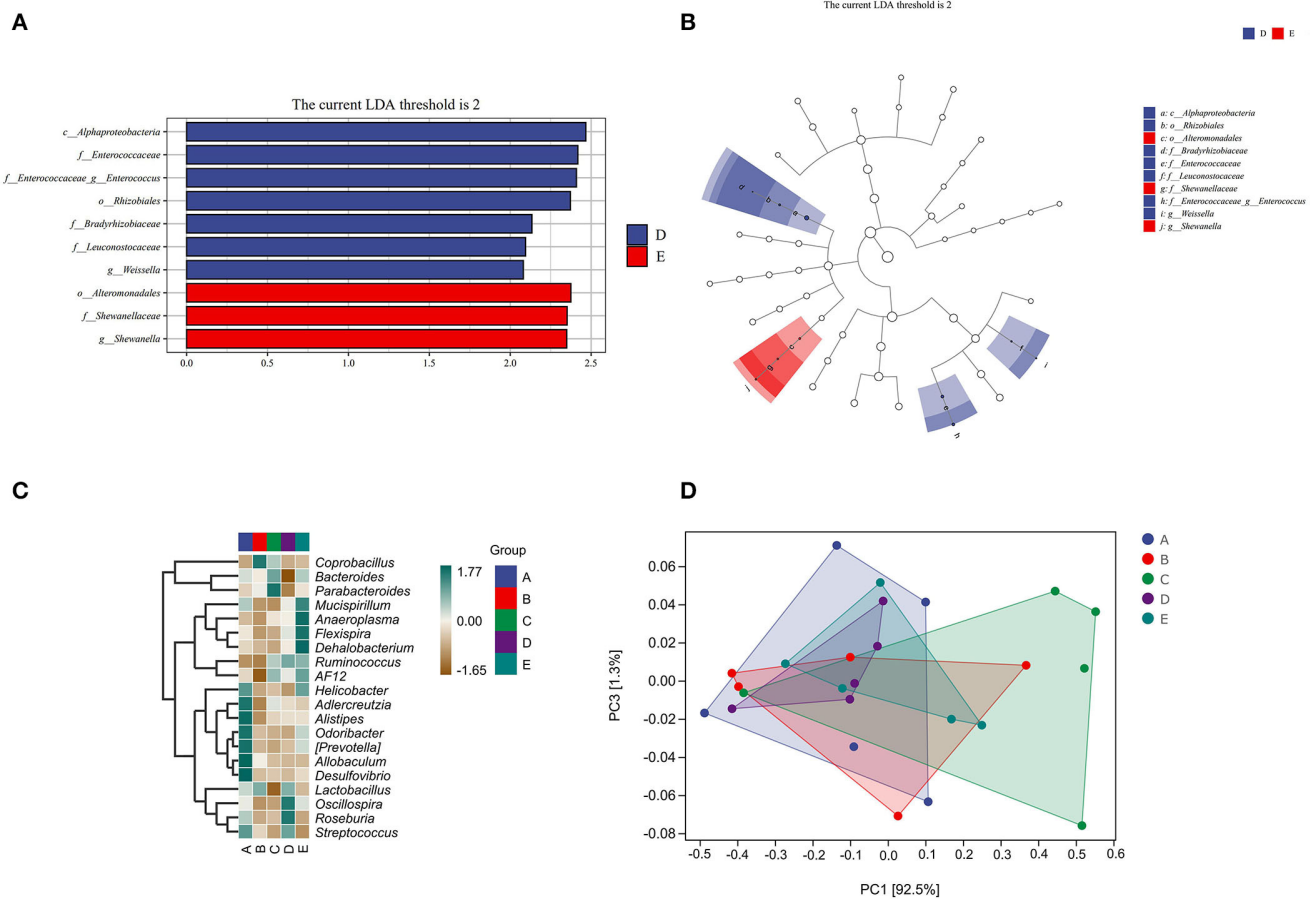
The effect of CSPCM on species differences and marker analysis in mice samples. **(A,B)** LEfSe analysis of intestinal flora. **(C)** Heat map of intestinal flora. **(D)** PCOA analysis of intestinal flora.

In Figures 6A,B, *Alphaproteobacteria* (at class level), *Enterococcaceae* (at the family level), *Enterococcus* (at genus level), *Rhizobiales* (at order level), *Bradyrhizobiaceae* (at the family level), *Leuconostocaceae* (at the family level), and *Weissella* (at genus level) in group D were significantly higher than that in other groups. *Alteromonadales* (at order level), *Shewanellaceae* (at the family level), and *Shewanella* (at genus level) in group E were significantly higher than that in other groups.

As shown in Figures 6C,D, *Helicobacter, Adlercreutzia, Alistipes, Odoribacter, Prevotella, Allobaculum, Desulfovibrio*, and *Streptococcus* were abundant in group A; *Coprobacillus* and *Lactobacillus* were abundant in group B; *Bacteroides* and *Parabacteroides* were abundant in group C; *Lactobacillus, Oscillospira, Roseburia*, and *Streptococcus* were abundant in group D; and *Mucispirillum, Anaeroplasma, Flexispira, Dehalobacterium, Ruminococcus, AF12*, and *Helicobacte*r were abundant in group E.

#### Analysis of CSPCM on intestinal flora composition in mice

**Figure 7:**
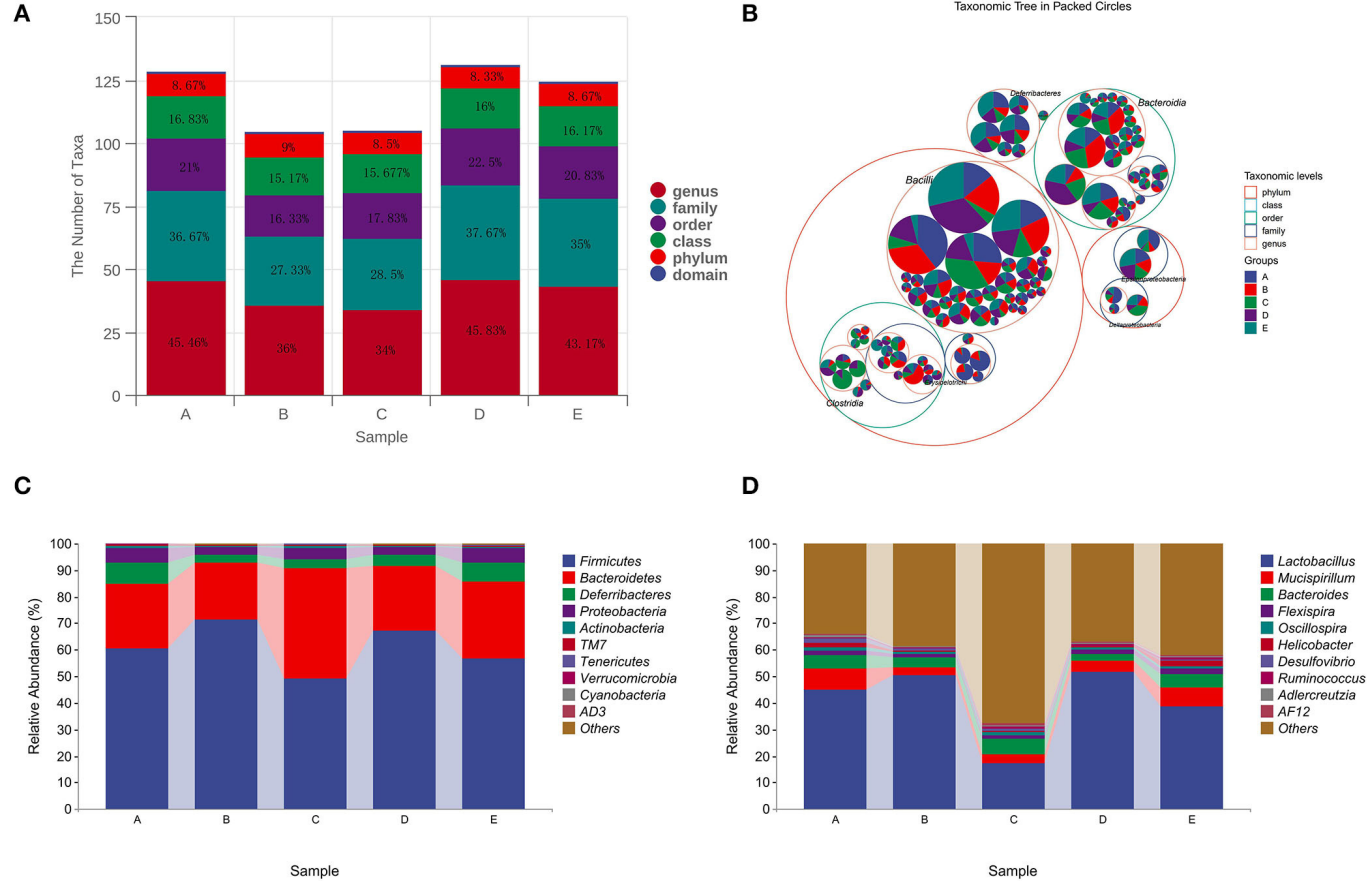
Analysis of CSPCM on intestinal flora composition in mice. **(A,B)** The effect of CSPCM on taxonomic composition in mice samples. **(C)** Effect of CSPCM on intestinal flora composition at the phylum level. **(D)** Effect of CSPCM on intestinal flora composition at the genus level.

Compared with group A, the number of taxa in group B decreased by 1.66, 4.67, 9.34, and 9.46% at class, order, family, and genus levels, respectively. After treatment with different doses of CSPCM, the number of taxa in group C increased by 0.507, 1.5, and 1.17% at class, order, and family levels, respectively, compared with group B. Compared with group B, the number of taxa in group D increased by 0.85, 7.53, 10.34, and 9.83% at class, order, family, and genus levels, respectively. Compared with group B, the number of taxa in group E increased by 1, 4.5, 7.67, and 7.17% at class, order, family, and genus levels, respectively (Figures 7A,B).

At the phylum level, *Firmicutes, Bacteroidetes, Deferribacteres*, and *Proteobacteria* were the most abundant bacteria. Compared with group A, the abundance of *Firmicutes* in group B increased, while the abundance of *Bacteroidetes, Deferribacteres*, and *Proteobacteria* decreased. Compared with groups B, C, D, and E, the abundance of *Firmicutes* decreased, while the abundance of *Bacteroidetes, Deferribacteres*, and *Proteobacteria* increased (Figure 7C).

At the genus level, *Lactobacillus, Mucispirillum, Bacteroides*, and *Flexisprra* were found to have high genus-level abundance. Compared with group A, the abundance of *Lactobacillus* in group B increased, while the abundance of *Mucispirillum, Bacteroides*, and *Flexisprra* decreased. Compared with groups B, C, D, and E, the abundance of *Lactobacillus* decreased, while the abundance of *Mucispirillum, Bacteroides*, and *Flexisprra* increased (Figure 7D).

## Discussion

The small intestine is the main organ of chemical digestion and absorption and the main absorption site of the protein, amino acids, starch, maltose, and glucose (Shi et al., 2017). The enhancement of the digestive function of the small intestine will contribute to the full absorption of nutrients. The small intestine is an important location for nutrient absorption, and increasing the length of villi in the small intestine can increase the contact area between the small intestine and intestinal contents, thus increasing the deposition of nutrients, which is of great significance for improving the health of animals (Wilson et al., 2018). Crypt depth reflects the colonization rate and maturity of crypt cells. The shallower the crypt, the better the maturity of cells, the better the secretion function, and the regeneration ability of villous epithelial cells, which is more conducive to maintaining intestinal absorption function. The longer the villi, the more contact with nutrients, and the more efficient the deposition of nutrients. The results show that the application of cyclophosphamide had a significant effect on the development of the small intestine, and the villi in each section of the small intestine of mice became sparse, and the villi length was significantly reduced. Meanwhile, cyclophosphamide inhibited the development and maturation of small intestinal crypts. It also indicates that the immunosuppressive model was successfully created. The therapeutic effect of CSPCM is obvious. In terms of morphology, CSPCM not only improves the thinning and shortening of villi in each section of the small intestine but also promotes the maturation of crypts. In terms of function, the use of CSPCM not only increased the contact area between the villi and the contents of the small intestine but also increased the secretory function of the crypt, so that the cyclophosphamide-treated mice returned to the normal intestinal absorption function.

The balance and stability of the immune system depend on the coordination of various immune organs, immune tissues, immune cells, and molecules and finally play a normal immune function. Pey's node is the immune induction site of the intestinal mucosal immune system, which is rich in thymogenic T cells, B cells, and DC immune cells, and the size and number of which can reflect the local immune status of intestinal mucosa. Lymphocytes in Pey's node are the main components of its external morphology, that is, the number of lymphocytes is closely related to its size. The cytotoxic effect of cyclophosphamide can induce the early apoptosis of parsonite node lymphocytes, thereby reducing the number of lymphocytes, and eventually leading to the atrophy and disappearance of Pey's node. In this experiment, CTX induced apoptosis, which resulted in the loosening of lymphocytes in Pey's node, while CSPCM could significantly ameliorate this situation, indicating that CSPCM can maintain the number and activation level of lymphocytes in animals and protect the normal morphology of Pey's node.

When the number and types of normal flora in the intestinal tract change, the lymphocytes in mesenteric lymph nodes can be rapidly activated and produce the corresponding cytokines to regulate the occurrence of immune responses. CD3+ lymphocytes are mainly divided into two types according to different antigens on the cell surface, namely, CD4+ cells and CD8+ cells. The results of this test show that CD3+, CD4+, and CD8+ cells were reduced by 97.3, 32.21, and 0.33%, respectively, compared with the normal group. In addition, it also caused a decrease in the ratio of CD4+/CD8+. CD3+ is a specific marker molecule on the surface of all T cells. Its level represents the level of the cellular immune response. The significant decrease in the proportion of CD3+ and CD4+ in group B indicates that cyclophosphamide greatly inhibits the body's cellular immunity.

Since CD4+ is a specific marker molecule on the surface of all helper lymphocytes (Th), this type of cell is activated by reacting with the polypeptide antigen presented by the MHCC-II molecular complex. CD4+ cells can secrete a variety of cytokines to promote the differentiation and proliferation of T and B cells. Therefore, cyclophosphamide also inhibits the secretion of cytokines IL-2, IL-22, and TNF-α, which ultimately leads to the disorder of the body's immune level. After CSPCM treatment, the proportion of CD3+, CD4+, and CD8+ cells in the mesenteric lymph nodes returned to normal levels, and subsequently, the levels of cytokines in the intestinal tract also increased and returned to normal levels. The mechanism of CSPCM's action lies in the enzymatic hydrolysate of astragalus in its ingredients. Astragalus is one of the best herbal medicines to replenish “qi.” The “qi” in traditional Chinese medicine theory can be approximately equal to the immunity in modern medicine. The study by Du has shown that *Astragalus membranaceus* can promote the maturation of dendritic cells in mice and inhibit Treg frequency to enhance immune response (Du et al., 2012). Li's study has shown that ginseng-astragalus-oxymatrine injection ameliorates cyclophosphamide-induced immunosuppression in mice (Li et al., 2021). Brush's study has shown that *Astragalus membranaceus* can activate immune cells in human subject. In addition, *Echinacea purpurea, Astragalus membranaceus*, and *Glycyrrhiza glabra* had an additive effect on the immune function when used in combination (Brush et al., 2006). The immune regulation effect of traditional Chinese medicine is to strengthen the body's resistance to consolidate the constitution, through the balance and stability of the immune system, to enhance the body's resistance to disease and reduce the purpose of pathogenic factors to the body damage.

In this study, LEfSe analysis, taxonomic analysis, and taxonomic composition analysis were used to evaluate the changes in the intestinal flora. Chinese medicine is metabolized by intestinal flora in the intestine, and the substances produced by the metabolism of intestinal flora affect the growth of intestinal flora. The results of intestinal flora composition analysis showed that cyclophosphamide changed the composition of intestinal flora, which was consistent with the existing research results (Kong et al., 2020; Xiang et al., 2021). After CSPCM treatment, the intestinal flora of mice tended to be normal. The statistical results of taxon number analysis showed that 100 g/kg bw CSPCM had no significant improvement on taxa number. The 0.2 g/kg·bw CSPCM and 0.4 g/kg·bw CSPCM significantly improved the taxon number at the level of class, order, family, and genus of cecum flora. The taxonomic composition analysis results were consistent with the statistical results of the taxonomic analysis. Cyclophosphamide changed the composition of cecal flora, increasing the proportion of *Fimicutes* and *Bacteroidetes* at the phylum level, increasing the proportion of *Lactobacillus*, and reducing the proportion of *Mucispirllum* at the genus level. The 0.1 g/kg·bw CSPCM did not prevent CTX from playing a destructive role in the intestine. The 0.2 g/kg·bw CSPCM and 0.4 g/kg·bw CSPCM played a certain role in the recovery of cecum intestinal flora. The 0.2 g/kg·bw CSPCM showed an excellent therapeutic effect, and the intestinal flora at phylum and genus levels returned to a normal state. LefSe analysis also showed that both 0.2 g/kg·bw CSPCM and 0.4 g/kg·bw CSPCM promoted the growth of specific bacterial communities in the cecum. As can be seen from the heat map results, the bacterial community of group B changed significantly, *Helicobacter, Adlercreutzia, Alistipes, Odoribacter, Prevotella, Allobaculum, Desulfovibrio*, and *Streptococcus* have a high abundance in the cecum of normal mice and a low abundance in group B. *Coprobacillus* and *Lactobacillus* had higher abundance in group B. The diversity of intestinal flora in group C was improved, the diversity and richness of cecal flora in groups D and E were significantly increased, and the intestinal flora in group E was more abundant, but the intestinal flora in group D was most similar to that in the control group. These results indicate that 0.2 g/kg·bw CSPCM had a better remodeling effect on cecal intestinal flora damaged by cyclophosphamide. The 0.4 g/kg·bw CSPCM supplementation also had a good effect on the remodeling of intestinal flora in immunosuppressed mice, which could increase its diversity and richness, but there were differences with the blank control mice in this experiment. It can be seen that 0.4 g/kg·bw CSPCM can promote the reconstruction of intestinal flora, but it will form a new intestinal flora compared with normal mice, and the role of this new intestinal flora remains to be further explored.

## Conclusion

CTX is extremely destructive to the intestinal area and has a great negative impact on the development of the small intestine, the intestinal immune system, and intestinal flora. CSPCM could ameliorate a series of negative effects on the intestinal region caused by CTX: restoring intestinal normal levels of IL-10, IL-17, IL-2, TNF-α, and sIgA; Increased numbers of CD3+, CD4+, and CD8+ cells in mesenteric lymph nodes. The villus length increased, crypt depth decreased, and the Pey's node area increased in all segments of the small intestine. At the phylum level, the intestinal flora was restored to normal by increasing the abundance of *Bacteroidetes, Deferribacteres*, and *Proteobacteria* and decreasing the abundance of *Firmicutes*. At the genus level, the gut microbiota was normalized by increasing the abundance of *Mucispirillum, Bacteroides*, and *Flexispira* and decreasing the abundance of *Lactobacillus*.

## Data availability statement

The datasets presented in this study can be found in online repositories. The names of the repository/repositories and accession number(s) can be found below: https://www.ncbi.nlm.nih.gov/, PRJNA822030.

## Ethics statement

The animal study was reviewed and approved by Ethics Committee of Animal Experiments of Heibei Agricultural University.

## Author contributions

YC: conceptualization ideas, methodology, and writing—original draft. WS and YB: project administration, funding acquisition. YJ: visualization. MD and CL: data curation. LZ: software, validation, and investigation. All authors contributed to the article and approved the submitted version.

## Funding

The study was supported by Graduate Student Innovation Ability Training funded project of Hebei Education Department, effects of compound small peptide of Chinese medicine on intestinal immunity and intestinal microflora in mice (CXZZBS2022044).

## Conflict of interest

Author YJ was employed by company Ringpu(baoding) Biological Pharmaceutical Co., Ltd. The remaining authors declare that the research was conducted in the absence of any commercial or financial relationships that could be construed as a potential conflict of interest.

## Publisher's note

All claims expressed in this article are solely those of the authors and do not necessarily represent those of their affiliated organizations, or those of the publisher, the editors and the reviewers. Any product that may be evaluated in this article, or claim that may be made by its manufacturer, is not guaranteed or endorsed by the publisher.
